# Trichostatin A-Assisted Epigenomic Modulation Affects the Expression Profiles of Not Only Recombinant Human α1,2-Fucosyltransferase and α-Galactosidase A Enzymes But Also Galα1→3Gal Epitopes in Porcine Bi-Transgenic Adult Cutaneous Fibroblast Cells

**DOI:** 10.3390/ijms22031386

**Published:** 2021-01-30

**Authors:** Jerzy Wiater, Marcin Samiec, Maria Skrzyszowska, Daniel Lipiński

**Affiliations:** 1 Department of Histology, Jagiellonian University Medical College, Kopernika 7 Street, 31-034 Kraków, Poland; 2 Department of Reproductive Biotechnology and Cryoconservation, National Research Institute of Animal Production, Krakowska 1 Street, 32-083 Balice n. Kraków, Poland; maria.skrzyszowska@izoo.krakow.pl; 3 Department of Biochemistry and Biotechnology, Poznań University of Life Sciences, Dojazd 11 Street, 60-647 Poznań, Poland; lipinskidaniel71@gmail.com

**Keywords:** pig, TSA-mediated epigenomic modulation, in vitro culture, bi-transgenic, adult cutaneous fibroblast cells, α1,2-fucosyltransferase, α-galactosidase A, Galα1→3Gal epitope, xenotransplantation

## Abstract

This study was conducted to explore whether trichostatin A-assisted epigenomic modulation (TSA-EM) can affect the expression of not only recombinant human α1,2-fucosyltransferase (rhα1,2-FT) and α-galactosidase A (rhα-Gal A) immune system enzymes but also Galα1→3Gal epitopes in ex vivo proliferating adult cutaneous fibroblast cells (ACFCs) derived from h*FUT2*×h*GLA* bi-transgenic pigs that had been produced for the needs of future xenotransplantation efforts. The ACFC lines were treated with 50 nM TSA for 24 h and then the expression profiles of rhα1,2-FT and rhα-Gal A enzymes were analyzed by Western blot and immunofluorescence. The expression profiles of the Galα1→3Gal epitope were determined by lectin blotting and lectin fluorescence. The ACFCs derived from non-transgenic (nTG) pigs were served as the negative (TSA^−^) and positive (TSA^+^) control groups. For both h*FUT2*×h*GLA* and nTG samples, the expression levels of α1,2-FT and α-Gal A proteins in TSA^+^ cells were more than twofold higher in comparison to TSA^−^ cells. Moreover, a much lower expression of the Galα1→3Gal epitopes was shown in TSA^−^ h*FUT2*×h*GLA* cells as compared to the TSA^−^ nTG group. Interestingly, the levels of Galα1→3Gal expression in TSA-treated h*FUT2*×h*GLA* and nTG ACFCs were significantly higher than those noticed for their TSA-untreated counterparts. Summing up, ex vivo protection of effectively selected bi-transgenic ACFC lines, in which TSA-dependent epigenetic transformation triggered the enhancements in reprogrammability and subsequent expression of h*FUT2* and h*GLA* transgenes and their corresponding transcripts, allows for cryopreservation of nuclear donor cells, nuclear-transferred female gametes, and resultant porcine cloned embryos. The latter can be used as a cryogenically conserved genetic resource of biological materials suitable for generation of bi-transgenic cloned offspring in pigs that is targeted at biomedical research in the field of cell/tissue xenotransplantation.

## 1. Introduction

Xenotransplantation seems to be a response to the contemporary shortage of organs for transplantation [1]. Choosing the right donor is difficult, but nowadays pigs are considered as the most suitable donors of tissues and organs for human patients with chronic or end-stage organ failure. Pigs share a large amount of similarities in organ anatomy and physiology; moreover, they share approximately 96% of their genetic identity with humans [2,3,4,5]. Unfortunately, the large phylogenetic distance between pig and human is the cause of the immunological barrier including immune response leading to hyperacute rejection (HAR), which currently makes pig-to-human of xenotransplantation unsuccessful [6]. The HAR is caused by the Galα1→3Gal epitope, which is present on the surface of porcine cells. The epitope Galα1→3Gal is formed by a reaction catalyzed by α1,3-galactosyltransferase (α1,3-GT), an enzyme encoded by the *GGTA1* gene. The α1,3-GT enzyme catalyzes the galactose transfer reaction from UDP-Gal and its α1→3 glycosidic bond with glycoproteins or glycosphingolipids containing terminal Galβ1→4GlcNAc-R residues [7]. One of the strategies to remove the Galα1→3Gal epitope from the porcine cells involves combined transgenic expression of recombinant human α1,2-fucosyltransferase (rhα1,2-FT) and α-galactosidase A (rhα-Gal A) enzymes encoded by h*FUT2* and h*GLA* genes, respectively [8]. Both endogenous porcine α1,3-galactosyltransferase and the introduced recombinant human α1,2-fucosyltransferase utilize N-acetyllactosamine. However, α1,2-FT is located in the intermediate compartment, and α1,3-GT in the trans part of the Golgi apparatus; therefore, α1,2-FT acts earlier than porcine endogenous α1,3-GT. As oligosaccharide moves through the Golgi apparatus it is first fucosylated by rhα1,2-FT, whereby it cannot accept the terminal galactose residue in subsequent reaction catalyzed by α1,3-GT [9]. In turn, the recombinant human α-galactosidase A cuts off the terminal D-galactose residues, and thus the Galα1→3Gal epitope becomes less xenoreactive [10]. Zeyland et al. [11] have shown the successful production of double-transgenic pigs with combined expression of α1,2-fucosyltransferase and α-galactosidase A. The above-mentioned investigators demonstrated that the co-expression of these two transgenes leads to a considerable reduction of the Galα1→3Gal epitope level on the surface of skin-derived fibroblast cells.

Thus far, various strategies of epigenomic modulation (epigenetic transformation) that are mediated by non-specific inhibitors of histone deacetylases (HDACi) and/or DNA methyltransferases (DNMTi) have been applied to improve reprogrammability of donor cell-inherited genome in somatic cell nuclear transfer (SCNT)-derived oocytes and resultant cloned embryos in different mammalian species [12,13,14,15,16]. Analogous methods of HDACi- and/or DNMTi-dependent epigenetic transformation have been adapted to enhance capabilities of parental genomes to be epigenetically reprogrammed in the in vitro fertilization (IVF)-derived embryos [17,18,19,20]. Such approaches have also been used either to expedite the epigenomic reprogramming and molecular dedifferentiation of adult somatic cells or mesenchymal stem cells (MSCs) into induced pluripotent stem cells (iPSCs) [21,22,23,24,25] or to facilitate the molecular rejuvenation of adult MSCs [21,26,27]. Moreover, the efforts that are aimed at the epigenomic modulation dependent on HDACi and/or DNMTi approved by the U.S. Food and Drug Administration (FDA) or by European Medicines Agency (EMA) have been undertaken to accomplish clinical treatments (i.e., epigenetic therapies) in oncologic patients and medical patients afflicted by neurodegenerative diseases or psychiatric disorders [28,29,30,31].

Taking into consideration the above-mentioned broad spectrum of practical applying exogenous epigenomic modulation, HDACi-assisted epigenetic transformation of in vitro-cultured somatic cells stemming from pigs genetically modified for the purposes of pig-to-human tissue xenotransplantation has not yet been reported. The scientific justification of such HDACi-dependently epigenomically modulating the genetically modified somatic cell lines is reflected in the finding that their treatment with non-selective HDACi designated as trichostatin A (TSA) can lead to improving reprogrammability of transgenes integrated with nuclear host genome. The molecular mechanism underlying improvements in the capabilities of the transgenes to be epigenetically reprogrammed in somatic cell nuclei encompasses direct enhancements in lysine moiety acetylation (i.e., hyperacetylation) processes of histones derived from chromatin nucleosomal cores associated with exon DNA sequences within promoters and/or enhancers of the transgenes that have been efficiently incorporated into host genome. TSA-dependent diminishments in histone deacetylation processes can indirectly trigger the enhancements in DNA cytosine residue demethylation (i.e., hypomethylation) reactions within promoter- and/or enhancer-related regions of the successfully integrated transgenes. These latter seem to evoke progressive onset and increase of transcriptional and translational activities of the incorporated transgenes and their mRNA transcript counterparts, respectively.

For all the reasons above, it is worth highlighting the fact that the effects of TSA-assisted epigenetic transformation of porcine double-transgenic fibroblast cell lines not only on the enzymatic expression levels of recombinant human α1,2-fucosyltransferase (α1,2-FT) and α-galactosidase A (α-Gal A) proteins but also on the semi-quantitative profiles of Galα1→3Gal antigenic determinants, i.e., epitopes, have been explored for the first time.

## 2. Results

### 2.1. Western Blot Analysis of the Relative Expression of Recombinant Human α1,2-Fucosyltransferase (rhα1,2-FT) and α-Galactosidase A (rhα-Gal A) Proteins

**Figure 1:**
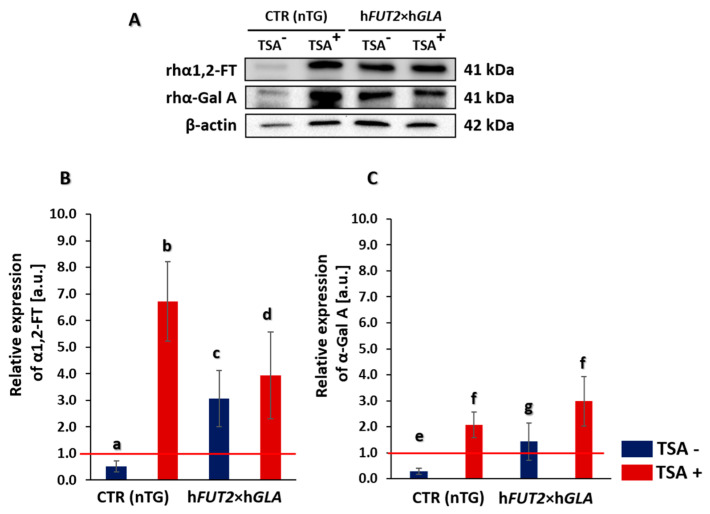
Western blot analysis of the relative expression of human α1,2-fucosyltransferase (α1,2-FT) and α-galactosidase A (α-Gal A) proteins in porcine bi-transgenic and non-transgenic adult cutaneous fibroblast cells (ACFCs) undergoing or not undergoing treatment with trichostatin A (TSA^+^ and TSA^−^, respectively). Representative blots of the expression of α1,2-FT and α-Gal A proteins in the ACFC samples derived from epigenetically modulated (TSA^+^) and non-modulated (TSA^−^) cells. The samples of non-transgenic animals served as a control group (CTR nTG—panel (**A**)). β-Actin served as a loading control for all analyzed samples. The results of relative expression (in arbitrary units) of α1,2-FT and α-Gal A are shown in panels (**B**,**C**), respectively. Relative optical density (ROD) from three separate analyses of at least three animals for each variant is expressed as mean. Bar graphs show mean ± standard error of the mean (SEM). The red line is taken as the cut-off value 1.0. Statistics: One-way ANOVA and Tukey’s honestly significant difference (HSD) post hoc test. The bars marked with different letters differ significantly; values denoted as a-b, a-d, b-c, b-d, e-f: *p* < 0.01; a-c, c-d, e-g, f-g: *p* < 0.05.

The adult cutaneous fibroblast cells (ACFCs) treated (TSA^+^) and non-treated with trichostatin A (TSA^−^) were derived from h*FUT2*×h*GLA* double-transgenic pigs and from non-transgenic pigs, which served as a control (CTR nTG). Western blot analysis of total protein samples showed the presence of recombinant human α1,2-fucosyltransferase (rhα1,2-FT) and α-galactosidase A (rhα-Gal A) proteins in all the transgenic samples (Figure 1A). A weak positive signal for both α1,2-FT and α-Gal A appeared in the TSA^−^ control group, but it was found to be non-significant. A clear positive signal was observed for both rhα1,2-FT and rhα-Gal A in total protein samples derived from TSA^+^ cells. Signal intensities of analyzed proteins were normalized to β-actin, which was used as a loading control. The semi-quantitative analysis of Western blot confirmed our observation. Indeed, the relative expression of both analyzed proteins was significantly higher (at least *p* < 0.05) in TSA^+^ cells as compared to the corresponding samples derived from TSA^−^ cells (Figure 1B). Interestingly, a clear positive signal was also identified for both α1,2-FT and α-Gal A proteins in TSA^+^ cells derived from non-transgenic pigs (CTR nTG). This result was confirmed both qualitatively and semi-quantitatively (at least *p* < 0.05) (Figure 1).

### 2.2. Immunofluorescence Localization of Recombinant Human α1,2-Fucosyltransferase (rhα1,2-FT) and α-Galactosidase A (rhα-Gal A) in Trichostatin A-Treated and Untreated ACFCs

**Figure 2:**
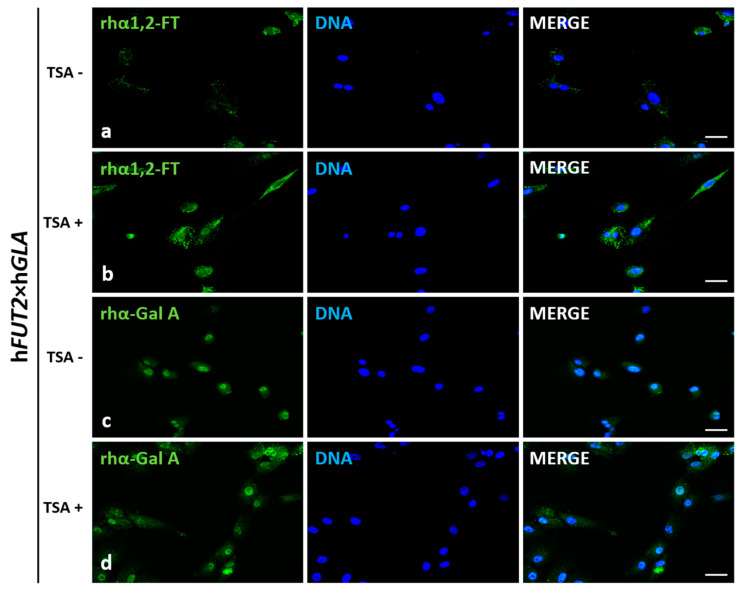
Immunofluorescence analysis of in vitro-cultured porcine ACFCs treated (TSA^+^) (**b**,**d**) and not treated with trichostatin A (TSA^−^) (**a**,**c**). Representative microphotographs of immunofluorescence localization of recombinant human α1,2-fucosyltransferase (rhα1,2-FT; **a**,**b**) and α-galactosidase A (rhα-Gal A; **c**,**d**) in ACFCs derived from double-transgenic pigs (h*FUT2*×h*GLA*). Immunofluorescent staining with Alexa Fluor 488-labelled secondary antibodies (green fluorescence) and 4′,6-diamidino-2-phenylindole (DAPI)-mediated counterstaining of cell nuclei (blue fluorescence). Scale bars represent 100 μm. Immunoreaction was performed on in vitro cultured porcine ACFCs from at least three pigs of each experimental group. The immunofluorescence signal stemming from rhα1,2-FT was distributed in the perinuclear region of all the analyzed cells from each variant. The recombinant human α-galactosidase A was located homogeneously in whole cytoplasm of all the analyzed cells. The TSA^+^ cells exhibited a more intense signal for both rhα1,2-FT and rhα-Gal A proteins (**b**,**d**) than their TSA^−^ counterparts (**a**,**c**).

**Figure 3:**
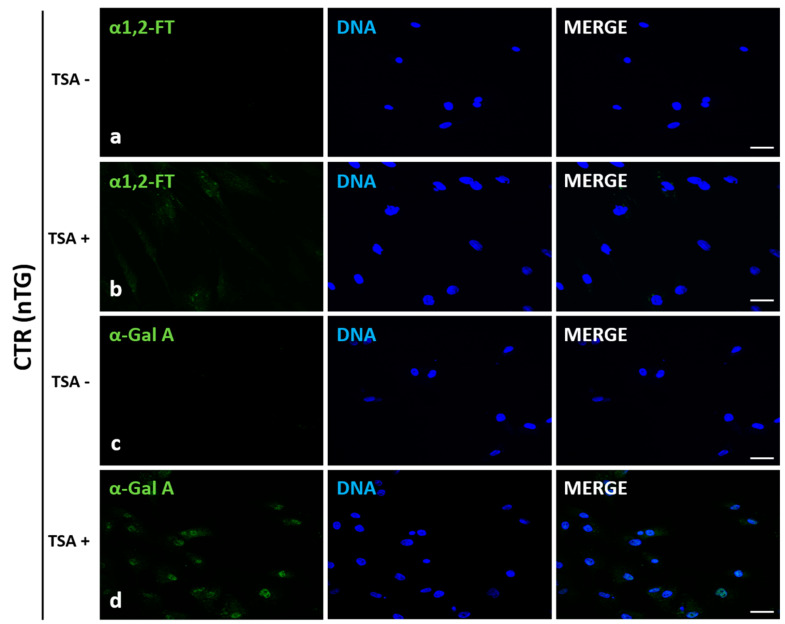
Immunofluorescence analysis of in vitro cultured porcine ACFCs treated (TSA^+^) (**b**,**d**) and not treated with trichostatin A (TSA^−^) (**a**,**c**). Representative microphotographs of immunofluorescence localization of human α1,2-fucosyltransferase (α1,2-FT; **a**,**b**) and α-galactosidase A (α-Gal A; **c**,**d**) in ACFCs derived from non-transgenic pigs (CTR nTG). Immunofluorescent staining with Alexa Fluor 488-labelled secondary antibodies (green fluorescence) and 4′,6-diamidino-2-phenylindole (DAPI)-mediated counterstaining of cell nuclei (blue fluorescence). Scale bars represent 100 μm. Immunoreaction was performed on in vitro-cultured porcine ACFCs from at least three pigs of each experimental group. The TSA^+^ cells exhibited a more intense signal for both α1,2-FT and α-Gal A proteins in control group (CTR nTG) (**b**,**d**). No positive signal stemming from α1,2-FT and α-Gal A in the TSA^−^ control (CTR nTG) was noticed (**a**,**c**).

Localization of recombinant human α1,2-fucosyltransferase (rhα1,2-FT) and α-galactosidase A (rhα-Gal A) was examined by immunofluorescence staining of trichostatin A-exposed (TSA^+^) and non-exposed (TSA^−^) ACFCs derived from h*FUT2*×h*GLA* double-transgenic (Figure 2) and non-transgenic (Figure 3) pigs utilized as a control (CTR nTG). The positive immunofluorescence signal descended from rhα1,2-FT was mainly distributed in small perinuclear clusters in bi-transgenic cells. In turn, rhα-Gal A immunostaining was found to be more homogenous and highly/intensively detectable in whole cytosol of transgenic (h*FUT2*×h*GLA*) cells (Figure 2). Clearly stronger immunofluorescence signal was observed for both rhα1,2-FT and rhα-Gal A proteins in cells treated with trichostatin A (TSA^+^) (Figure 2b,d). Moreover, a weak immunofluorescence signal for both α1,2-FT and α-Gal A was also identified in non-transgenic (CTR nTG) TSA^+^ cells (Figure 3b,d). However, in the TSA^−^ CTR nTG cells, the α1,2-FT was barely detectable by immunofluorescence, while any positive signal descended from α-galactosidase A was not identified (Figure 2).

### 2.3. Lectin Blotting Analysis of Galα1→3Gal Epitope Expression at the Protein Level in the In Vitro-Cultured Porcine Bi-Transgenic and Non-Transgenic ACFCs Treated and Not Treated with Trichostatin A

**Figure 4:**
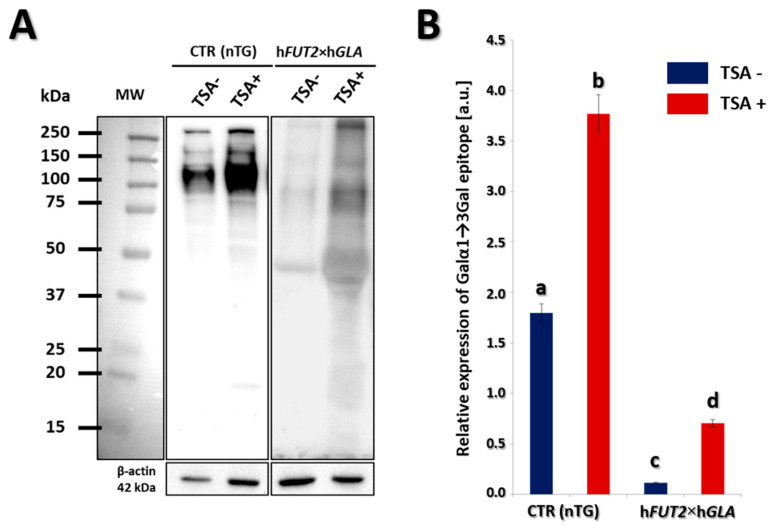
Lectin blotting analysis of Galα1→3Gal epitope expression at the protein level in the in vitro cultured porcine transgenic and non-transgenic ACFCs treated (TSA^+^) and not treated with trichostatin A (TSA^−^). (**A**) Representative lectin blots of the expression of Galα1→3Gal epitope in TSA^−^ and TSA^+^ in vitro-cultured ACFCs derived from non-transgenic (CTR nTG) control and double-transgenic (h*FUT2*×h*GLA*) pigs. MW indicates the molecular weight of protein standards (Precision Plus Protein Dual Color Standards, Bio-Rad). Each band represents a glycosylated protein containing the Galα1→3Gal epitope. β-Actin served as a loading control for all analyzed samples. (**B**) The semi-quantitative analysis of Galα1→3Gal epitope relative expression (in arbitrary units). Relative optical density (ROD) from three separate analyses of at least three animals for each variant is expressed as mean. Graph bar shows mean ± SEM. Statistics: One-way ANOVA and Tukey’s HSD post hoc test. The bars marked with different letters differ significantly. Values denoted as a-b, a-c, a-d, b-c, b-d: *p* < 0.01; c-d: *p* < 0.05. The relative expression of Galα1→3Gal epitope was significantly lower in the ACFCs stemming from h*FUT2*×h*GLA* bi-transgenic pigs as compared to the control (CTR nTG) non-transgenic animals. It is also noteworthy to highlight the fact that the TSA^−^ cell samples derived from h*FUT2*×h*GLA* double-transgenic pigs were characterized by the remarkably lowest expression of Galα1→3Gal epitopes. However, expression of Galα1→3Gal epitopes was significantly higher in TSA^+^ cell samples compared to that identified for their TSA^−^ counterparts derived from both transgenic and non-transgenic pigs.

Lectin blot analysis was used to determine the expression profile of Galα1→3Gal epitopes at the total protein level using horseradish peroxidase (HRP)-conjugated lectin GS-IB_4_. The results of our study confirmed that significantly lower expression of Galα1→3Gal epitopes was identified in bi-transgenic fibroblast cells as compared to the control group. In turn, both in control (CTR nTG) and double-transgenic (h*FUT2*×h*GLA*) groups, expression of Galα1→3Gal epitopes was found to be significantly increased in the cell samples derived from trichostatin A (TSA)-treated ACFCs (TSA^+^; Figure 4A). β-Actin served as a loading control. The quantitative analysis of h*FUT2*×h*GLA* TSA confirmed these observations. The significantly lowest expression of Galα1→3Gal epitopes was detected in TSA-untreated (TSA^−^) ACFCs derived from h*FUT2*×h*GLA* double-transgenic pigs, transpiring to statistically differ from both CTR nTG TSA^−^ (*p* < 0.01) and CTR nTG TSA^+^ groups (*p* < 0.01). The expression of Galα1→3Gal epitopes in ACFCs originating from h*FUT2*×h*GLA* TSA^+^ group was significantly higher (*p* < 0.05) than that noticed for cells stemming from h*FUT2*×h*GLA* TSA^−^ group, but it was found to still be lower than that indicated in both CTR nTG groups (*p* < 0.01) (Figure 4B).

### 2.4. Identification of the Expression Profiles of Galα1→3 Gal Epitope by Fluorescently Labelled Lectin GS-IB4

**Figure 5:**
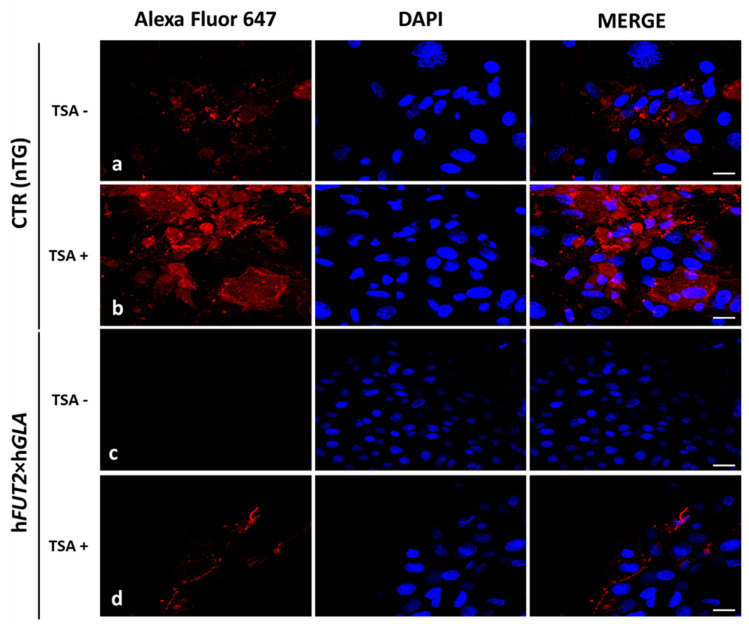
Lectin fluorescence analysis of Galα1→3Gal epitope expression in the in vitro-cultured porcine transgenic and non-transgenic ACFCs treated (TSA^+^) (**b**,**d**) and not treated with trichostatin A (TSA^−^) (**a**,**c**). Representative microphotographs of lectin GS-IB_4_ labelled sections derived from non-transgenic control (CTR nTG; **a**,**b**) and double-transgenic (h*FUT2*×h*GLA*; **c**,**d**) pigs. The lectin fluorescence analysis was performed with the use of Alexa Fluor 647-conjugated lectin GS-IB_4_ (red fluorescence) and 4′,6-diamidino-2-phenylindole (DAPI)-mediated counterstaining of cell nuclei (blue fluorescence). Scale bars represent 50 μm.

For identifying the expression profiles of Galα1→3 Gal epitope, we used lectin GS-IB4 labelled with Alexa Fluor 647 (Figure 5). The lectin GS-IB_4_ strongly stained Galα1→3 Gal antigenic determinants in trichostatin A-treated (TSA^+^) ACFCs originating from the CTR nTG group (Figure 5b). In contrast, TSA^+^ and TSA^-^ ACFCs stemming from h*FUT2*×h*GLA* double-transgenic pigs displayed a much lower Galα1→3Gal fluorescence intensity as compared to both TSA^+^ and TSA^−^ control groups. However, in h*FUT2*×h*GLA* group, we observed stronger Galα1→3Gal fluorescence intensity in TSA^+^ ACFCs compared to their TSA^−^ counterparts (Figure 5c,d).

## 3. Discussion

The hyperacute rejection is the main obstacle in pig-to-human xenotransplantation. To overcome the immune response, genetically modified pigs must be generated. Research around the world shows different ways of genetic modification and their combinations. These include generating homozygous pigs lacking the gene for α1,3-galactosyltransferase for the purpose of depletion of anti-pig antibodies, removal of the Galα1→3Gal epitope using enzymes, and engineering pigs transgenic for certain graft-protective proteins [32,33,34,35].

In the current study, we investigated the effects of epigenetic modulation by trichostatin A on overexpression of recombinant human α1,2-fucosyltransferase and α-galactosidase A, and on the amount of Galα1→3Gal antigen in the porcine ACFCs derived from bi-transgenic and non-transgenic lines. Since genetically modified pigs were produced to avoid hyperacute rejection [12,36,37], we discuss this aspect in relation to an adult cutaneous fibroblast cell (ACFC)-based in vitro model. Our Western blot and immunofluorescence analyses revealed the significant enhancements in the expression of both recombinant human α1,2-fucosyltransferase and α-galactosidase A proteins in the epigenomically modulated ACFCs (TSA^+^) derived from h*FUT2*×h*GLA* bi-transgenic pigs as compared to their TSA^−^ cell counterparts. Moreover, immunofluorescence staining with antibodies against human α1,2-fucosyltransferase and α-galactosidase A provided evidence that the epigenetic modulation with TSA led to increase in the expression α1,2-fucosyltransferase and α-galactosidase A proteins in ACFCs. The study by Jia et al. [38] confirmed that α-galactosidase alone reduced the expression of Galα1→3Gal antigenic determinants by 78%, while the co-expression of both α1,2-FT and α-Gal A diminished the quantitative profile of these epitopes to a negligible level on the surface of SV40 immortalized aortic porcine endothelial cells. In turn, the previous investigation by Wiater et al. [39] has proven that co-expression of recombinant human α-galactosidase A and α1,2-fucosyltransferase decreased the expression of Galα1→3Gal antigenic determinants by 62% (on the basis of histochemistry) and 47% (on the basis of blotting), respectively. However, in the previously mentioned studies, relatively low expression of rhα1,2-FT and rhα-Gal A was demonstrated, which could be the reason for the lack of complete silencing the expression of Galα1→3Gal epitopes. Therefore, the use of epigenomic modulation of the cells to increase expression of these proteins seems to be inevitable. It is also worth noting that the impacts of TSA-dependent epigenetic transformation of porcine bi-transgenic ACFCs not only on the biocatalytic expression levels of recombinant human α1,2-fucosyltransferase (rhα1,2-FT) and α-galactosidase A (rhα-Gal A) proteins but also on the semi-quantitative profiles of Galα1→3Gal epitopes have been investigated for the first time. Indeed, in the current study, we showed by Western blot analyses that TSA-mediated epigenomic modulation of ACFCs led to increase in the relative expression of both rhα1,2-FT and rhα-Gal A by 28.32% and 108.96%, respectively. In our present study, we demonstrated by semi-quantitative (lectinblotting) and qualitative (lectin fluorescence) methods that the expression of Galα1→3Gal antigenic determinants in porcine ACFCs was significantly declined in cells derived from h*FUT2*×h*GLA* double-transgenic pigs. The efficiency of Galα1→3Gal epitope reduction in cells not treated with TSA remained at the level of 93.63%, while in TSA^+^ cells, at the level of 81%. Moreover, Western blot analyses showed that the relative expression of Galα1→3Gal epitopes increased by 109.83% in non-transgenic (CTR nTG) TSA^+^ cells compared to the TSA^−^ cells. Thus, these results provided proof that trichostatin A-assisted epigenetic transformation affects not only expression profiles of rhα1,2-FT and rhα-Gal A proteins but also relative abundance of Galα1→3Gal antigenic determinants in ACFCs.

To sum up, we have shown that the overexpression of both recombinant human α1,2-fucosyltransferase and α-galactosidase A in a porcine h*FUT2*×h*GLA* bi-transgenic ACFCs model significantly reduced but did not eliminate the Galα1→3Gal epitope from the surface of cells. Moreover, we have shown that the treatment of ACFCs with non-selective HDACi designated as trichostatin A (TSA) can lead to improvement in the reprogrammability of transgenes integrated with nuclear host genome, but possibly also the host’s own genes. Therefore, taking into account cell/tissue xenotransplantation, it is clear that the most effective way to eliminate Galα1→3Gal epitopes is by producing homozygous α-1,3-galactosyltransferase knock-out (GTKO) pigs lacking the gene for α1,3-galactosyltransferase.

## 4. Materials and Methods

### 4.1. In Vitro Culture and Trichostatin A-Mediated Epigenomic Modulation of Fibroblast Cells

The adult cutaneous fibroblast cell (ACFC) lines were established according to the protocols described in our previous studies [40,41,42]. In our current investigation, ACFCs derived from h*FUT2*×h*GLA* bi-transgenic pigs (n = 3) were utilized [11]. The ACFCs stemming from non-transgenic pigs were served as a control group (CTR nTG). All animal procedures that were used in the study by Zeyland et al. [11] were conducted in accordance with the European Directive 2010/63/EU and approved by the Second Local Ethics Committee in Kraków, Poland (Permission 1181/2015 from 21st May 2015). All ACFC lines were cultured in Dulbecco’s Modified Eagle’s Medium/Nutrient Ham’s Mixture F-12 (DMEM/F-12) (1:1) (Sigma-Aldrich, St. Louis, MO, USA) supplemented with 15% fetal bovine serum (FBS; Sigma-Aldrich) and 1% penicillin/streptomycin cocktail (Sigma-Aldrich) in CO_2_ incubator under the stabilized conditions as follows: temperature of +38.5 °C, 5% CO_2_, and relative humidity (RH) of air atmosphere ranging from 90 to 95%. For Western and lectin blot analyses, cells were cultured in T-25 flasks up to 2–3 passage, but for immunofluorescence, the cells at the second passage were seeded onto sterile coverslips in 6-well plates. When the ex vivo-expanded ACFC lines reached approximately 85% of confluence, their epigenomic modulation was initiated by adding 50 nM of trichostatin A (TSA; Sigma-Aldrich) to the culture medium. Both bi-transgenic and non-transgenic fibroblast cells were epigenetically transformed by exposure to TSA for 24 h.

### 4.2. Total Protein Extraction and Western/Lectin Blot Analyses

Total protein was extracted from harvested ACFCs by using radioimmunoprecipitation assay lysis buffer (RIPA buffer, Thermo Fisher Scientific, Waltham, MA, USA) containing 1% of proteinase inhibitor cocktail (RIPA+PI; Bioshop Inc., Burlington, VT, Canada). After TSA treatment, the cells were washed with phosphate-buffered saline (PBS; Biomed, Lublin, Poland) and then 300 μL of RIPA+PI was added per flask, and cells were harvested with cell scrapers. The samples were subsequently sonicated and centrifuged at 13,200 rpm for 15 min at +4 °C and supernatant collected. Protein concentration was estimated with microassay DC Protein Assay (Bio-Rad Laboratories, Hercules, CA, USA) using bovine serum albumin (BSA) as a standard. Protein samples were stored at −80 °C for further analyses.

For SDS-PAGE, protein samples were diluted in 2× Laemmli Sample Buffer (Bio-Rad Laboratories, Hercules, CA, USA) containing β-mercaptoethanol and denaturated at 99.5 °C per 5 min. Electrophoresis was performed with 5% stacking and 10% resolving polyacrylamide gels. Each lane was loaded with 20 μg of protein. Then, proteins were electro-transferred onto poly(vinylidene fluoride) (PVDF) membrane (Immobilon-P; Merck, Darmstadt, Germany) at constant amperage 250 mA for 120 min.

For immunoblotting, membranes after several washes in Tris-buffered saline (TBS) were blocked for 1 h in 5% non-fat milk in TBST (Tris-buffered saline with 0.1% *v*/*v* Tween20; Bioshop Inc., Burlington, VT, Canada). Then, membranes were washed several times in TBST and incubated overnight at +4 °C with the following primary antibodies: against human α1,2-fucosyltransferase (diluted 1:1000 in TBST; rabbit polyclonal antibodies, ab198712, Abcam, Cambridge, UK) and human α-galactosidase A (diluted 1:1000 in TBST; rabbit polyclonal antibodies, PA5-27349, ThermoFisher Scientific, Waltham, MA, USA). β-Actin was served as a loading control protein (diluted 1:2000 in TBST; mouse monoclonal antibodies, ab8224, Abcam). Then, membranes were washed several times in TBST and incubated with goat anti-rabbit or goat anti-mouse horseradish peroxidase (HRP)-conjugated secondary antibodies (ThermoFisher Scientific, Waltham, MA, USA) at a dilution 1:6000 in TBST for 1 h at room temperature.

For lectinblotting, membranes were blocked for 30 min in 1% BSA (Bioshop Inc., Burlington, Canada) in TBST. Then, membranes were washed three times in Dulbecco’s phosphate-buffered saline (DPBS) containing Ca^2+^/Mg^2+^ ions (Gibco, ThermoFisher Scientific, Waltham, MA, USA) followed by TBS. In the next step, membranes were incubated overnight at +4 °C with lectin GS-IB_4_ labelled with HRP (L5391, Sigma-Aldrich) diluted 1:2000 in DPBS. Finally, membranes were washed in TBS buffer.

For both Western blotting and lectin blotting, protein bands were detected by chemiluminescence using Clarity Western ECL Blotting Substrate (Bio-Rad Laboratories, Hercules, CA, USA) and visualized with the ChemiDoc XRS+ Imaging System (Bio-Rad Laboratories, Hercules, CA, USA). Protein bands were quantified using the Image Lab 2.0 Software (Bio-Rad Laboratories, Hercules, CA, USA). Semi-quantitative analysis was performed for 3 separately repeated experiments for each control and experimental group and normalized on reference protein (i.e., β-actin)-related signal. Each analysis was calculated as follows:

(1) $$Relative\;expression=\frac{signal_{SAMPLE}}{signal_{REFERENCE\;PROTEIN}}$$

Subsequently, the results encompassing the relative expression of the analyzed proteins (i.e., rhα1,2-FT and rhα-Gal A enzymes) were shown as a mean ± standard error of the mean (SEM).

### 4.3. Immunofluorescence Staining

The ACFCs after TSA treatment were washed with sterile PBS and fixed with 4% paraformaldehyde for 10 min at room temperature (RT). After several washes in PBS, cells were blocked in 5% normal goat serum (NGS) in PBST (PBS containing 0.1% Triton X-100) for 30 min. Cells were then incubated overnight at +4 °C in humidified chamber with the following primary antibodies (the same as those for Western blot): against human α1,2-fucosyltransferase (diluted 1:150 in PBST) and human α-galactosidase (diluted 1:200 in PBST). In the next step, cells were washed several times in PBST and treated with goat anti-rabbit Alexa Fluor 488-conjugated secondary antibodies (diluted 1:300 in PBST; ThermoFisher Scientific, Waltham, MA, USA) for 1 h at room temperature. After final washes, cells were mounted in Fluoroshield with 4′,6-diamidino-2-phenylindole (DAPI) mounting medium (F6057, Sigma-Aldrich). Fluorescently labelled ACFCs were examined as described in Section “*4.5. Confocal microscope analyses*”.

### 4.4. Lectin Fluorescence

For intracellular localization of Galα1→3Gal epitopes and consequent semi-quantitative comparison of their expression profiles between TSA-treated and untreated dermal fibroblast cells originating from h*FUT2*×h*GLA* and non-transgenic (CTR nTG) pigs, we used lectin from *Griffonia simplicifolia* (GS-IB_4_) conjugated with Alexa Fluor 647 fluorescent dye (I32450, Molecular Probes, Invitrogen, ThermoFisher Scientific, Waltham, MA, USA). The ACFCs after TSA treatment were washed with sterile PBS and fixed with 4% paraformaldehyde for 10 min at RT. After several washes in PBS, cells were blocked in 1% BSA in PBST for 1 h. Cells were subsequently rinsed thrice in PBS and treated with lectin GS-IB_4_ diluted 1:200 in DPBS at +4 °C overnight in a dark humidified chamber. After final washes, cells were mounted in Fluoroshield with DAPI and coverslipped. Fluorescently labelled ACFCs were examined as described in Section “*4.5. Confocal microscope analyses*”.

### 4.5. Confocal Microscope Analyses

Fluorescently labelled cells were examined by the confocal microscope Olympus FluoView 1200 on inverted stand IX83 (Olympus, Tokyo, Japan). Forty-times magnification objective (NA = 0.95) was used, and diode laser (473 nm), diode laser (635 nm), and diode laser (405 nm) were applied to excite green (Alexa Fluor 488), far-red (Alexa Fluor 647), and blue (DAPI) fluorescence, respectively.

### 4.6. Statistical Analysis

For each TSA^+^ or TSA^−^ cell derived from genetically modified and non-modified pigs and for all analyses, we performed three repeats. Quantitative data were expressed as the mean ± standard error of the mean (SEM) and examined using the Shapiro–Wilk *W* test for normality. Comparisons between the appropriate means were performed by one-way analysis of variance (ANOVA), followed by Tukey’s honestly significant difference (HSD) post hoc test for multiple ranges. All statistical analyses were accomplished using Statistica 13 Software (StatSoft Inc., Tulsa, OK, USA). Statistical significance was marked by letters at the appropriate charts. The bars marked with different letters differ significantly.

## 5. Conclusions

The ex situ conservation of efficiently selected (desirable) double-transgenic adult cutaneous fibroblast cell (ACFC) lines, in which TSA-mediated epigenomic modulation has resulted in the improved reprogrammability and thereby increased transcriptional and translational activities of h*FUT2* and h*GLA* transgenes and their counterpart mRNA transcripts, enables us to cryogenically preserve reservoirs of nuclear donor somatic cells, nuclear-transferred oocytes reconstructed with somatic cells, and corresponding cloned pig embryos. These biorepositories of somatic cell lines, nuclear-transferred female gametes, and resultant embryos can provide a source of cryopreserved biological materials that are reliable and feasible for research targeted at producing bi-transgenic somatic cell nuclear transfer (SCNT)-derived piglets for the purposes of pig-to-human cell and/or tissue xenograft transplantation.

It is noteworthy to highlight that irrespective of the mechanism, by which TSA-mediated epigenomic modulation of ACFCs brings about increase in the relative abundance for Galα1→3Gal epitopes, the extent of Galα1→3Gal expression decreased remarkably in both TSA-treated and untreated h*FUT2*×h*GLA* bi-transgenic ACFCs displaying overabundance of rhα1,2-FT and rhα-Gal A enzymes as compared to their non-transgenic counterparts.

Finally, further detailed research is required to focus on not only transcriptomic and proteomic profiling but also recognizing the molecular mechanisms underlying TSA-mediated enhancement of the relative abundance for Galα1→3Gal epitopes in porcine h*FUT2*×h*GLA* double-transgenic ACFCs and subsequently their potential use for procedures of cloning pigs by SCNT.

## Abbreviations

ACFCs	Adult cutaneous fibroblast cells
DNMTi	Inhibitors of DNA methyltransferases
HAR	Hyperacute rejection
HDACi	Inhibitors of histone deacetylases
HRP	Horseradish peroxidase
iPSCs	Induced pluripotent stem cells
MSCs	Mesenchymal stem cells
rhα1,2-FT	Recombinant human α1,2-fucosyltransferase
rhα-Gal A	Recombinant human α-galactosidase A
SCNT	Somatic cell nuclear transfer
TSA	Trichostatin A
α1,3GT	α1,3-galactosyltransferase
